# Rapid Motion Adaptation Reveals the Temporal Dynamics of Spatiotemporal Correlation between ON and OFF Pathways

**DOI:** 10.1038/srep34073

**Published:** 2016-09-26

**Authors:** Can Oluk, Andrea Pavan, Hulusi Kafaligonul

**Affiliations:** 1 National Magnetic Resonance Research Center (UMRAM), Bilkent University, Ankara, Turkey; 2 Department of Psychology, Bilkent University, Ankara, Turkey; 3 University of Lincoln, School of Psychology, Brayford Pool, Lincoln, LN6 7TS, UK; 4 Interdisciplinary Neuroscience Program, Bilkent University, Ankara, Turkey

## Abstract

At the early stages of visual processing, information is processed by two major thalamic pathways encoding brightness increments (ON) and decrements (OFF). Accumulating evidence suggests that these pathways interact and merge as early as in primary visual cortex. Using regular and reverse-phi motion in a rapid adaptation paradigm, we investigated the temporal dynamics of within and across pathway mechanisms for motion processing. When the adaptation duration was short (188 ms), reverse-phi and regular motion led to similar adaptation effects, suggesting that the information from the two pathways are combined efficiently at early-stages of motion processing. However, as the adaption duration was increased to 752 ms, reverse-phi and regular motion showed distinct adaptation effects depending on the test pattern used, either engaging spatiotemporal correlation between the same or opposite contrast polarities. Overall, these findings indicate that spatiotemporal correlation within and across ON-OFF pathways for motion processing can be selectively adapted, and support those models that integrate within and across pathway mechanisms for motion processing.

At the early stages of visual analysis, two major categories of retinal ganglion cells have been identified based on their contrast polarity preferences: ON- and OFF-center ganglion cells. ON-center cells are stimulated by a luminance increment at the center of their receptive fields whereas OFF-center cells are stimulated by a luminance decrement. The neural signals originating in the ON- and OFF-center retinal ganglion cells remain segregated in the LGN up to the initial stages of the primary visual cortex. Signals from ON and OFF pathways are merged by complex cells in the striate cortex^1^,^2^,^3^,^4^,^5^.

In the motion domain, a well-known phenomenon demonstrating the perceptual consequence of combining ON and OFF signals is the reverse-phi illusion^6^,^7^. In the reverse-phi illusion, the contrast polarity is reversed at each spatial displacement. Unlike in regular motion (i.e., no polarity change), the direction of reverse-phi motion is perceived in the direction opposite to the physical displacement. The perceived direction of the reverse-phi motion is consistent with the outcome of motion energy model^8^ and the equivalent Reichardt detector^9^. It has been demonstrated that the spatial and temporal limits for regular and reverse-phi motion are comparable^10^,^11^ and sensitivity for both motion types are similar^12^. Moreover, adapting to reverse-phi motion induces a motion aftereffect (MAE) comparable to that obtained when adapting to regular motion, suggesting that reverse-phi motion adaptation is likely to reduce the activity of low-level motion sensors^13^. In fact, there is physiological evidence in cats and macaque monkeys that reverse-phi motion activates directional selective neurons in primary visual cortex (V1) and MT tuned to the direction opposite to the physical displacement^14^,^15^. Though these studies describe the neural correlates of reverse-phi motion at early stages of motion processing, it is still unclear how spatiotemporal correlation between opposite contrast polarities is achieved for directional selectivity^13^,^16^.

In order to understand how signals from the two pathways are combined for reverse-phi motion direction selectivity, Mo *et al.*^17^ carried out simulations indicating that computational models based on strictly separate processing of ON and OFF signals (e.g., asymmetric delayed-inhibition model by Barlow *et al.*^18^) can easily account for regular motion selectivity, but not for reverse-phi motion selectivity. Additionally, they found that a direct interaction between ON and OFF pathways is essential to achieve spatiotemporal correlation between opposite contrast signals and reverse-phi directional selectivity. Though this approach incorporating within and across pathway mechanisms has been supported by many psychophysical studies on motion perception, there are different views on how the signals from the two pathways are combined. Some of the behavioral findings emphasize the segregation of ON and OFF pathways up to the local motion stage and suggest an effective correlation mechanism between two pathways prior to the extraction of the global motion signal^19^. On the other hand, relatively recent studies emphasize across pathway mechanisms at low-level motion detection stage^13^,^17^,^20^.

Kanai *et al.*^21^ found that even a brief exposure to motion can induce adaptation, biasing the perceived direction of a subsequently presented ambiguous (i.e., counterphase flickering) test stimulus. By using brief adaptation durations and adaptation-test blank intervals (i.e., inter-stimulus interval; ISI), Kanai *et al.*^21^ and other following studies^22^,^23^,^24^,^25^ showed that the perceived direction of an ambiguous test pattern can be biased towards the opposite direction (rapid motion aftereffect – rMAE) or towards the same direction (rapid visual motion priming – rVMP) to that of the adapting pattern^26^. Besides, for adaptation durations of a few hundred milliseconds (e.g., 320 ms) and a sufficiently long ISIs (e.g., 2 s), the perceived direction of the ambiguous test pattern is again biased towards the same direction to that of the adapting pattern. Such prolonged and long-lasting effect is called Perceptual Sensitization (PS). These distinctive effects of rapid motion adaptation are considered to be perceptual manifestations of neural plasticity at different levels of motion processing^21^,^23^,^24^,^27^,^28^.

Due to these adaptation effects observed over different time scales, the rapid motion adaptation paradigm provides a fruitful approach to assess the temporal dynamics of the neural mechanisms involved in motion processing. In this study, we took advantage of the rapid motion adaptation paradigm in order to assess the across pathway mechanisms for motion processing and to compare them with the motion mechanisms within each pathway. In particular, we adapted observers to regular motion (probing motion processing within ON and OFF pathways) and reverse-phi motion (probing motion processing across the two pathways). Test patterns were counterphase flickering (i.e., directionally ambiguous) stimuli either engaging spatiotemporal correlation within or across pathways. We systematically examined the dynamics of rapid motion adaptation across these different experimental conditions.

## Experiment 1

We used an adapting stimulus either probing spatiotemporal correlation between the same or opposite contrast polarities upon each spatial displacement, and measured its effect on a subsequently presented ambiguous test pattern with a specific contrast polarity. By varying the duration of the adapting stimulus and the adapting-test blank interval (i.e., inter-stimulus interval; ISI), we were able to compare the temporal dynamics of mechanisms based on the spatiotemporal correlation within and across ON-OFF pathways.

### Methods

#### Participants

One of the authors (CO) and fourteen naïve observers (age range: 20–27) participated in Experiment 1. Observers had normal or corrected-to-normal visual acuity. Viewing was binocular. Participants gave informed consent, and all procedures were in accordance with international standards (Declaration of Helsinki, 1964) and approved by the ethics committee at Ankara University.

#### Apparatus

We used Matlab version 7.12 (The MathWorks, Natick, MA) with Psychtoolbox 3.0^29^,^30^ for stimulus presentation and data acquisition. Visual stimuli were presented on a 20-inch CRT monitor (HP p1230, 1280 × 1024 pixel resolution and 85 Hz refresh rate) at a viewing distance of 57 cm. The minimum and maximum luminance of the screen were 0.36 and 98.95 cd/m^2^, respectively. Luminance was measured with a SpectroCAL (Cambridge Research Systems, Rochester, Kent, UK) photometer. A gamma-corrected lookup table (LUT) was used so that luminance was a linear function of the digital representation of the image. Head movements were constrained by a chin rest. All experiments were performed in a dark room.

#### Stimuli and Procedure

**Figure 1:**
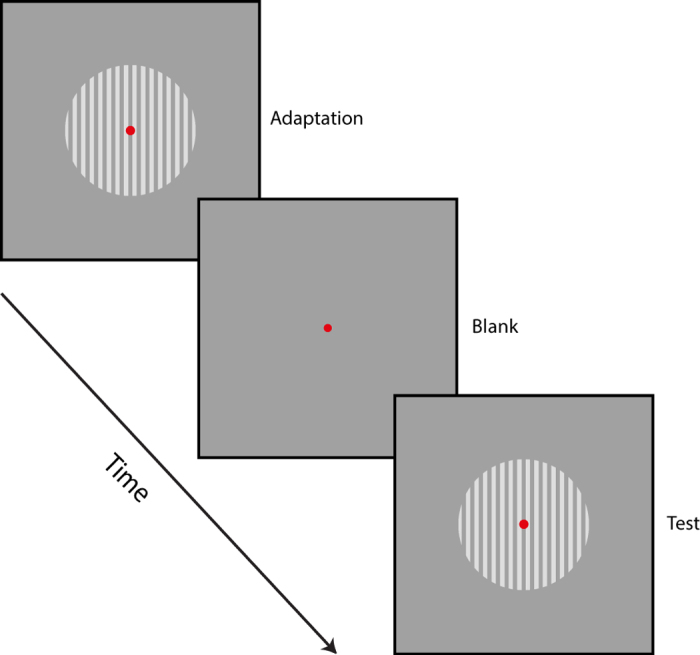
Representation of stimuli and motion adaptation sequence used in all the experiments. First, the adapting stimulus was shown. The motion direction of the adapting pattern was either leftward or rightward, and its duration was varied across trials. After a variable adapting-test blank interval (i.e., inter-stimulus interval; ISI), a directionally ambiguous test grating was displayed for 752 ms.

**Figure 2:**
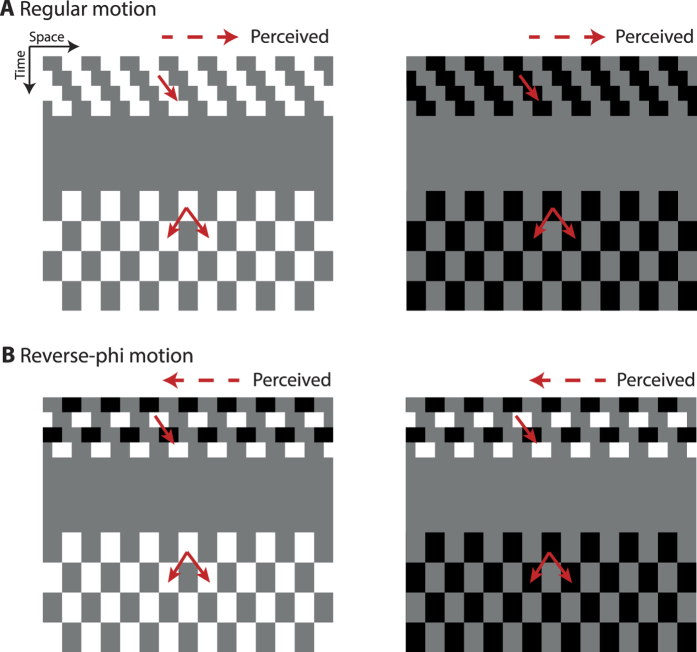
Space-time representation of the stimuli used in Experiment 1. The horizontal and vertical axes in the space-time plots correspond to the horizontal spatial axis and time, respectively. In the examples shown, the phase of the adapting grating was shifted from left-to-right and only one adaptation duration (356 ms) and one blank interval (482 ms) are represented. (**A**) The perceived motion direction for regular motion was in the direction of the physical displacement (left-to-right). (**B**) Contrast reversal in consecutive frames (i.e., in every 90 deg phase shift) was introduced for reverse-phi motion. Such change in contrast polarity led to motion percept in the direction opposite (right-to-left) to the physical displacement. Both motion types were followed by directionally ambiguous test stimuli either brighter or darker than the background. During an experimental session, only one motion type (regular or reverse-phi motion) was used as adapting stimulus.

A small red circle (12 arc-min diameter) at the center of the display served as a fixation target. Adapting stimuli were vertically oriented drifting square-wave gratings with spatial frequency of 1 cpd (Fig. 1). The gratings were viewed through a circular aperture (radius: 8 deg) centered on the screen. The duty cycle was 50% and the luminance of the half period was equal to background luminance (16 cd/m^2^). In order to create motion in horizontal direction, the phase of the grating was shifted by ±90 degrees (left or right) every motion frame. We used two types of motion (regular and reverse-phi) as adapting stimuli. For regular motion, the gratings were either lighter (27 cd/m^2^) or darker (5 cd/m^2^) than the gray background throughout the presentation of motion (Fig. 2A). We used the same luminance values for reverse-phi motion. However, instead of having fixed luminance throughout the motion sequence, the contrast polarity of the grating was reversed in each of the two consecutive frames (Fig. 2B). The duration of each motion frame was 94 ms and there was no temporal interval between each frame for both motion types.

The test stimulus was created by using the same square-wave gratings. As in regular motion, we used either lighter or darker patterns throughout the duration of test stimulus (752 ms). However, the phase was shifted by 180 degrees every 188 ms (Fig. 2). This manipulation led to directionally ambiguous dynamic (i.e., counterphase flickering) test patterns with speed matching that of the adapting stimulus (2.66 deg/s).

We used a similar procedure to that of Kanai *et al.*^21^ and Pavan *et al.*^24^. During each trial, observers fixated on the red circle at the center of the display. Adapting stimulus (square-wave grating drifting either rightward or leftward) was first presented for a duration chosen pseudorandomly from three values: 188, 376 and 752 ms. After a variable ISI (i.e., 35, 118, 482, 1000, and 2000 ms), during which only the fixation point was present, the counterphase flickering test pattern was displayed for 752 ms. At the end of each trial, observers were requested to indicate, by a key press, whether the test pattern moved in the same or opposite direction as compared to the adapting stimulus. When observers were adapted to regular motion, the contrast polarity of the adapting and test patterns was the same (Fig. 2A). For reverse-phi motion adaptation, the contrast polarity of the test pattern and the starting polarity (i.e., contrast polarity of the first frame) of each motion sequence were pseudorandomly selected from the two polarity options (Fig. 2B). However, the starting phase of the test pattern (relative to the adapter) was not randomized for both motion adaptation types. Each experimental condition (3 adaptation durations × 5 ISIs) was presented 12 times per session. Accordingly, there were 180 trials in each experimental session. Regular and reverse-phi motion adaptation were run in separate sessions and the order of these sessions was randomized across observers. Each observer completed 2 sessions for each motion type, and hence a total of 24 trials were collected (from each observer) for each data point.

As shown by many studies, reverse-phi motion is perceived in the direction opposite to the physical displacement^10^,^11^,^12^. In order to assess whether the observers perceived all the adapting stimuli in the “expected direction”, each observer also participated in a control experiment prior to the main experiment described above. In this control experiment, only the adapting stimulus was presented. Observers indicated by a key press whether the adapting stimulus moved leftward or rightward (method of single stimuli: MSS^31^). Each control session included a balanced mixture of the two motion types (i.e., regular and reverse-phi motion) and had 6 experimental conditions (3 stimulus durations × 2 motion types). Every condition was presented 36 times per session. For regular motion, the adapting stimulus was brighter than the background for half of the trials and darker than the background for the rest of the trials. Each observer completed one control session. Observers who could identify the direction of reverse-phi motion patterns in the expected direction (i.e., opposite to the physical displacement) more than 75% of the trials for all conditions were included in the main experiment. All the fifteen participants reported above perceived motion in the expected direction more than 75% for all adaptation durations and motion types.

### Results and Discussion

**Figure 3:**
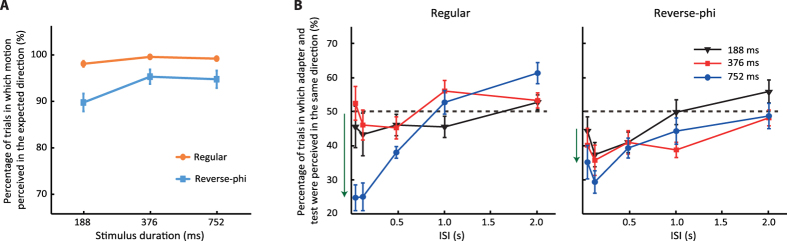
Results of Experiment 1 (N = 15). (**A**) Motion direction reports for regular and reverse-phi motion as a function of duration. The “expected” direction for regular and reverse-phi refers to the same and opposite directions with respect to the physical displacement. (**B**) Rapid motion adaptation results. For each adaptation duration, the percentage of trials in which observers judged the test stimulus as drifting in the same direction to that of the adapting pattern is shown as a function of the ISI. The results for each motion type are shown in separate plots. The green arrows near the ordinate highlight the changes in adaptation curves due to the increment of adaptation duration. Error bars ± SEM.

As shown in Fig. 3A, regular and reverse-phi motion types were reliably perceived as moving in the “expected direction” for all stimulus durations. In other words, the perceived direction of regular motion was consistent with the physical displacement of the grating in more than 95% of the trials for all stimulus durations. On the other hand, the reverse-phi motion was perceived in the direction opposite to physical displacement. When compared to regular motion reported in the physical direction, the proportion of responses in the opposite direction for reverse-phi motion was slightly lower (~5%). The difference between the two motion types became greater for the shortest motion duration (~8%). A two-way repeated measures ANOVA with the motion type (regular and reverse-phi) and the stimulus duration as factors, reported a significant effect of motion type (*F*_1,14_ = 11.07, *p* = 0.005, *partial-η*^2^ = 0.442) and a significant interaction between motion type and duration (*F*_2,28_ = 3.45, *p* = 0.046, *partial-η*^2^ = 0.198). Bonferroni corrected pairwise comparisons between the two motion types for each duration showed a significant difference for 188 ms (*p* = 0.001) and 356 ms (*p* = 0.028), but only a marginal significant difference for 752 ms (*p* = 0.054).

Figure 3B shows the results of the main experiment. For all adaptation durations, the percentage of trials in which the directionally ambiguous test pattern was perceived to drift in the same direction to that of the adapting pattern was plotted as a function of ISI. A three-way repeated measures ANOVA including the motion type, adaptation duration and ISI as factors, reported a significant effect of adaptation duration (*F*_2,28_ = 7.55, *p* = 0.002, *partial-η*^2^ = 0.35), ISI (*F*_4,56_ = 10.654, *p* < 0.001, *partial-η*^2^ = 0.432) and a marginally significant effect of the motion type (*F*_1,14_ = 4.43, *p* = 0.054, *partial-η*^2^ = 0.240). The two-way interaction between motion type and adaptation duration (*F*_2,28_ = 6.55, *p* = 0.005, *partial-η*^2^ = 0.319) and the three-way interaction between motion type, adaptation duration and ISI were significant (*F*_8,112_ = 6.56, *p* < 0.001, *partial-η*^2^ = 0.319). To understand the exact nature of these interactions, we performed additional repeated measures ANOVA (adaptation duration and ISI as factors) on each motion type separately. For regular motion, we found a significant effect of adaptation duration (*F*_2,28_ = 19.47, *p* < 0.001, *partial-η*^2^ = 0.582) and a significant interaction between adaptation duration and ISI (*F*_8,112_ = 12.29, *p* < 0.001, *partial-η*^2^ = 0.468). However, for reverse-phi motion, the effect of adaptation duration (*F*_2,28_ = 2.83, *p* = 0.076, *partial-η*^2^ = 0.168) and the interaction between adaptation duration and ISI were not significant (*F*_8,112_ = 1.51, *p* = 0.161, *partial-η*^2^ = 0.097). Furthermore, additional two-way ANOVA tests on each adaptation duration indicated a significant interaction between motion type and ISI (*F*_4,56_ = 8.84, *p* < 0.001, *partial-η*^2^ = 0.387) only for the longest adaptation duration (i.e., 752 ms). Bonferroni corrected post-hoc pairwise comparisons between the two motion types at individual ISI conditions of the longest adaptation duration suggested that this interaction were mostly due to the significant stronger rMAE at 118 ms of ISI (*p* = 0.038) and stronger priming effects at the longer ISI values for regular motion (ISI = 1 s, *p* = 0.046; ISI = 2 s, *p* = 0.001).

In order to further assess the temporal characteristics of rapid VMP and MAE obtained with the two motion types, we also performed a series of one-sample *t-*tests to test whether each combination of adaptation duration and ISI was significantly different from chance level (i.e., 50%). Results are summarized in Table 1. Though none of the motion types led to a significant rapid visual priming (rVMP), they induced significant rapid motion aftereffects (rMAEs) (Table 1). For the shortest adaptation duration, both motion types had similar effect on the ambiguous test pattern either brighter or darker than background luminance. As regular motion, reverse-phi motion induced rMAE (and even found to be more effective according to the statistical testing shown in Table 1) for ISI values less than 500 ms. The rMAE was decreased for longer ISI values. As the adaptation duration was increased, the adaptation induced by both motion types differed. Reverse-phi motion led to almost similar adaptation curves (i.e., similar dependency on ISI) for all adaptation durations. On the other hand, the influences of regular motion on the test pattern became much more effective when the adaptation duration was 752 ms. For this adaptation duration, the regular motion induced strong rMAE for short ISI values (i.e., 35 and 118 ms). However, as the ISI was further increased, the regular adaptation curve quickly approached to the baseline (i.e., 50% chance level) and the perceived direction of test pattern was biased towards the same direction as the adapting pattern for the longest ISI values (PS). Only the priming effect with regular motion for 2 s ISI condition was found to be significantly different from chance (Table 1). This suggests that PS can be induced only by regular motion when both adaptation duration and ISI are long.

Accumulating evidence suggests that asymmetries in response dynamics of ON and OFF pathways can lead to differences in the perception of bright (positive contrast) and dark (negative contrast) visual stimuli^32^,^33^,^34^. To test whether such differences in processing dynamics can be observed in the rapid motion adaptation curves, we separately analyzed the regular motion trials containing stimuli brighter and darker than the background. For regular motion, we did not find a significant effect of contrast polarity but its interaction with adaptation duration was significant (for ANOVA test results see Supplementary Table S1). Follow-up tests revealed that this interaction was mostly due to the differences between the two contrast polarities when the adaptation duration was 752 ms (Supplementary Table S2). For this adaptation duration, the percentage of trials in which the bright test pattern was perceived in the same direction to that of the bright adapting stimulus was higher than the dark conditions (Supplementary Fig. S1). In other words, the bright conditions of regular motion (Fig. 2A) led to slightly less motion aftereffect and higher priming than the dark conditions.

Additionally, we tested whether reverse-phi motion adaptation had different influences on bright and dark test patterns (Fig. 2B). Even though the interaction between contrast polarity and adaptation duration was significant (Supplementary Tables S3 and S4), the adaptation effects did not reveal a consistent difference as in regular motion (Supplementary Fig. S2). In fact, we did not find any significant difference between the rapid effects obtained by testing with bright or dark patterns. These results suggest that reverse-phi motion is likely to adapt both ON and OFF pathways. In our experiments, the starting phase of the test pattern was not randomized relative to the adapter. This may lead to an apparent motion between the last frame of the reverse-phi and the first frame of the test pattern at short ISI values. In this case, we expect the same and opposite polarity pairs of the last reverse-phi frame and the first test frame should result in regular and reverse-phi apparent motion (Fig. 2B). To this purpose, we analyzed the opposite and same polarity conditions separately (i.e., the contrast polarity combinations of the last frame of the reverse-phi and the first frame of the test pattern). However, our analyses did not reveal any consistent difference between these two conditions (for statistical analyses see Supplementary Material).

Overall, these findings suggest that reverse-phi motion may lead to similar adaptation curves to those obtained with regular motion. However, the amount of the adaptation effects on the subsequently presented directionally ambiguous test pattern having a constant contrast polarity (i.e., mostly engaging spatiotemporal correlation between the same contrast polarity), have distinct dependencies on adaptation duration for each motion type. In particular, for longer adaptation durations (e.g., 752 ms), regular motion with a fixed contrast polarity led to stronger adaptation effects on the test pattern having the same contrast polarity. On the other hand, the adaptation effects induced by reverse-phi motion did not show a significant dependency on adaptation duration. These differences in the temporal dynamics of rapid adaptation cannot be simply explained in terms of different saliency (and/or strength) of the two motion types. As shown in Fig. 3A, the difference in motion direction discrimination for the two motion types is bigger at the shortest stimulus duration and it gets smaller as the stimulus duration increases. Such a dependency on duration would imply that any perceived difference in motion salience (and/or strength) should also be bigger at the shortest duration. If any motion salience (and/or strength) led to the observed changes in behavioral results, the adaptation curves for these motion types should be significantly different than each other when the adaptation duration is short. However, this is not case for the shortest adaptation duration. Any significant difference between the adaptation curves is mostly observed when the adaptation duration becomes longer (e.g., 752 ms).

## Experiment 2

In Experiment 1, test stimuli were directionally ambiguous gratings either brighter or darker than the background. It is expected that such test stimulus mostly engage spatiotemporal correlations within the same channel. In Experiment 2, we used test stimuli changing contrast polarity at each spatial displacement, and hence engaging spatiotemporal correlations between opposite contrast polarities. Since reverse-phi motion has been considered to mostly engage spatiotemporal correlations between opposite polarities, the reverse-phi adaptation should be more effective on the test pattern used in the present experiment. Therefore, the rapid effects should be more pronounced for reverse-phi adaptation than regular motion when the adaptation duration becomes longer.

### Methods

#### Participants

One of the authors (CO) and eleven naïve observers (age range: 20–23) took part in Experiment 2. Five of these observers completed Experiment 1.

#### Stimuli and Procedure

**Figure 4:**
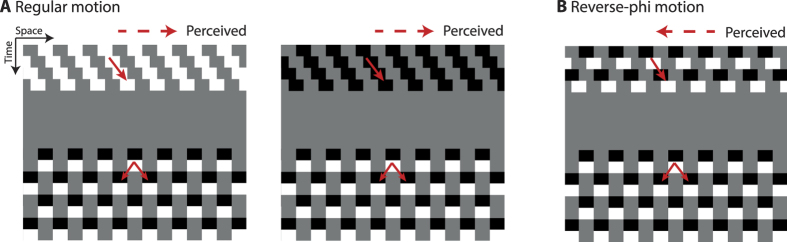
Space-time representation of the stimuli used in Experiment 2. The contrast polarity of the test stimulus was changed every 94 ms. The rate of polarity change matched that of the reverse-phi motion and the contrast polarity was reversed when there was a 180 deg phase shift.

Instead of using test stimuli having a fixed contrast polarity, we introduced a contrast polarity change every 94 ms of the test motion sequence. As in the previous experiment, the spatial phase was shifted by 180 degrees every 188 ms (Fig. 4). These temporal characteristics of the test pattern resulted in both a temporal frequency for contrast polarity change matching that of the reverse-phi motion (5.32 Hz) and a reversal in contrast polarity upon each spatial displacement. Therefore, the dynamic flickering test pattern engaged spatiotemporal correlation between two opposite polarities. For each motion adaptation type, the contrast polarity of the test pattern and the starting polarity (contrast polarity of first frame) of each motion sequence were pseudorandomly selected from two polarity options. However, the starting phase of the test pattern (relative to the adapter) was not randomized. Except these changes introduced in the ambiguous test stimuli, all other stimulus parameters, conditions and experimental procedure were the same as those used in Experiment 1.

### Results and Discussion

**Figure 5:**
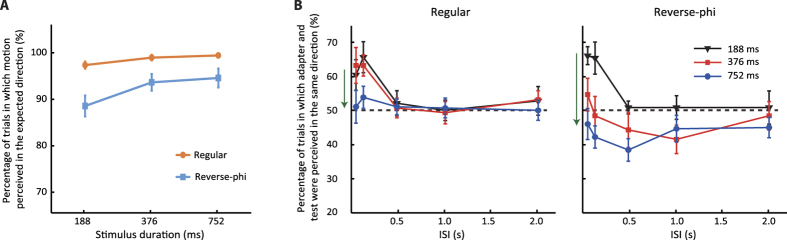
Results of Experiment 2 (N = 12). (**A**) Motion direction reports for regular and reverse-phi motion as a function of stimulus duration. (**B**) Rapid motion adaptation results. For each adaptation duration, the percentage of trials in which observers judged the test stimulus as drifting in the same direction to that of the adaptation stimulus is shown as a function of the ISI. The green arrows near the ordinate highlight the changes in adaptation curves due to the increment of adaptation duration. Error bars ± SEM.

The results of the control session were similar to those of the previous experiment. As shown in Fig. 5A, the regular and reverse-phi motion led to motion percepts in the same and opposite direction to that of the physical displacement, respectively. The regular motion direction reports in the physical direction were slightly higher than those in the reverse direction for reverse-phi. These differences were higher for the lower stimulus durations (i.e., 188 and 376 ms). A repeated measures ANOVA with the motion type and stimulus duration as factors, reported a significant effect of motion type (*F*_1,11_ = 10.21, *p* = 0.009, *partial-η*^2^ = 0.481) and a significant interaction between motion type and stimulus duration (*F*_2,22_ = 3.99, *p* = 0.033, *partial-η*^2^ = 0.266). Bonferroni corrected pairwise comparisons showed that direction reports in the expected direction for regular motion were significantly higher than reverse-phi for all stimulus durations (188 ms, *p* = 0.003; 376 ms, *p* = 0.019; 752 ms, *p* = 0.049).

Figure 5B shows the adaptation results for both motion types. A repeated measures ANOVA including the motion type, adaptation duration and ISI as factors, reported a significant effect of adaptation duration (*F*_2,22_ = 15.38, *p* < 0.001, *partial-η*^2^ = 0.583) and ISI (*F*_4,44_ = 6.30, *p* < 0.001, *partial-η*^2^ = 0.364), but not a significant effect of the motion type (*F*_1,11_ = 3.087, *p* = 0.107, *partial-η*^2^ = 0.219). However, the interaction between motion type and adaptation duration was significant (*F*_2,22_ = 11.64, *p* < 0.001, *partial-η*^2^ = 0.514). To assess the nature of this interaction, a two-way repeated measures ANOVA (motion type and ISI as factors) was performed for each adaptation duration. Though we did not find a significant difference between the two motion types for the 188 ms adaptation duration (*F*_1,11_ = 0.022, *p* = 0.88, *partial-η*^2^ = 0.002), the motion type was found to be significant for longer adaptation durations (i.e., 376 ms adaptation duration: *F*_1,11_ = 5.051, *p* = 0.046, *partial-η*^2^ = 0.315; 752 ms adaptation duration: *F*_1,11_ = 8.17, *p* = 0.016, *partial-η*^2^ = 0.426).

Moreover, we performed separate two-way repeated measures ANOVA (adaptation duration and ISI as factors) for each motion type. The ANOVA reported a significant effect of adaptation duration for both regular (*F*_2,22_ = 5.60, *p* = 0.011, *partial-η*^2^ = 0.337) and reverse-phi motion adaptation (*F*_2,22_ = 18.76, *p* < 0.001, *partial-η*^2^ = 0.630). However, its interaction with ISI was significant only for reverse-phi motion (*F*_8,88_ = 2.12, *p* = 0.042, *partial-η*^2^ = 0.161). Additionally, we performed statistical tests on the bright and dark conditions of regular motion. The results indicated no significant effect of contrast polarity and its interactions with other factors (Supplementary Table S5). As in Experiment 1, we also analyzed the effect of contrast polarity combinations of the last frame of the adapter and the first frame of the test pattern. Our analyses did not reveal any consistent difference between the two conditions for both motion adaptation types (for statistical analyses see Supplementary Material).

As shown in Fig. 5B, both motion types led to similar adaptation curves for the shortest adaptation duration (188 ms). Contrary to Experiment 1, adaptation to both motion types produced significant rapid visual priming effects (Table 2) for short ISI values. Increasing the adaptation duration further resulted in losing rVMP effects for both regular and reverse-phi motion, but had also distinct effects on regular and reverse-phi adaptation curves. In support of the ANOVA results above, Bonferroni-corrected pairwise comparisons pointed out some differences between regular and reverse-phi adaptation curves when the adaptation duration was longer than 188 ms. For 376 ms adaptation duration, there was a significant difference between two motion types at 118 ms of ISI (*p* = 0.016). For the longest adaptation duration (752 ms), the two motion types were significantly different for both 118 ms (*p* = 0.024) and 482 ms (*p* = 0.005) of ISIs. Reverse-phi motion also led to a significant rMAE at 482 ms of ISI when the adaptation duration was 752 ms (Table 2).

The results here point out differences in the dynamics of regular and reverse-phi motion adaptation, and they complement the results of Experiment 1. Adaptation to both motion types had significant effects on the directionally ambiguous dynamic test pattern engaging spatiotemporal correlation between opposite contrast polarities. However, as the adaptation duration was increased, the rapid priming effect induced by regular motion decayed and only adaptation to reverse-phi motion induced reliable rMAEs at intermediate ISI values. As in Experiment 1, it is unlikely that these observed differences in dynamics can be simply explained in terms of motion salience and/or strength differences between the two motion types. The percentage of directional reports in the expected direction for regular motion was significantly higher than reverse-phi motion. Such a difference implies that observers perceived the direction of regular motion better than reverse-phi. Hence, based on these differences between the two motion types, we would expect that the regular motion adaptation should have been more effective for all adaption durations. However, overall, our findings suggest more dominant adaptation effects induced by reverse-phi motion.

### General Discussion

One of the most fundamental segregations of visual signals is achieved through separate ON and OFF pathways selectively responding to contrast increments and decrements, respectively. There is physiological evidence that these two pathways remain mostly segregated until primary visual cortex and merged at the level of V1 complex cells^1^,^2^,^4^. Several studies emphasized the segregation of the two pathways at early stages of visual motion processing, suggesting that the information carried by the two pathways remains segregated and feeds separate motion detectors^19^,^35^. On the other hand, the spatiotemporal correlation between opposite polarities and the integration of information from the two pathways has an essential role in motion perception. Sensitivity measurements on motion types engaging spatiotemporal correlation between ON and OFF pathways, suggest the presence of an efficient detection mechanism, similar to those responding to stimuli that probe within-pathway mechanisms^10^,^12^. In the current study, we investigated the dynamics of within and across pathway mechanisms by adapting observers to moving stimuli mostly probing spatiotemporal correlations between the same (regular motion) and opposite (reverse-phi motion) contrast polarities. Our results showed that both motion types led to similar adaptation curves for short adaptation durations. However, for the longest adaptation duration (752 ms), regular motion engaging spatiotemporal correlation between same polarities produced much stronger adaptation effects on the test pattern having the same contrast polarity (Experiment 1). On the other hand, Experiment 2 showed that the longest adaptation to reverse-phi motion (752 ms) had a significantly stronger influence on the test pattern engaging spatiotemporal correlations between opposite polarities. These differences in adaptation curves for both motion types cannot be explained by differences in motion salience and/or strength. It has been pointed out that such adaptation effects are built-up over time in distinct information processing channels selectively activated through motion adaptation^36^. Therefore, the dissociation between regular and reverse-phi motion can be explained by selectively adapting either spatiotemporal correlation within or across ON-OFF pathways first and then, activating the same mechanism through corresponding ambiguous test patterns.

#### Rapid Forms of Motion Adaptation

Our findings demonstrate for the first time that adapting to reverse-phi motion may lead to rapid visual motion priming (rVMP) and motion aftereffect (rMAE). Accumulating evidence supports the notion that rVMP and rMAE reflect facilitation (potentiation) and suppression (inhibition) at early stages of motion processing^21^,^24^. The extent to which these adaptation effects can be modulated using moving stimuli is thought to reflect distinct motion processes. For instance, rVMP has been found to be completely absent when adapting to moving stimuli engaging later stages of motion processing^27^. Therefore, in conjunction with previous studies on reverse-phi motion^12^,^14^, the existence of rVMP and rMAE induced by reverse-phi motion adaptation also supports the view that information provided by ON and OFF pathways is effectively combined at early stages of motion processing.

The differences in the dynamics of spatiotemporal correlation within each and across the two pathways are obvious in our study. For instance, when the spatiotemporal correlation between the same pathways was adapted by regular motion for 752 ms and then selectively activated by a test pattern with the same polarity, the observed rMAE was stronger at the shorter ISI values and decreased with increasing the ISI (Fig. 3B). However, when observers were adapted to reverse-phi motion and subsequently judged the direction of a test pattern engaging spatiotemporal correlations between two pathways, the induced rMAE was stronger for relatively longer ISI values (Fig. 5B). Based on this difference, we argue that the rMAE from spatiotemporal correlation between the two pathways, requires more time to develop than the rMAE induced by stimuli engaging the same pathway. Moreover, the rVMP was found to be absent when testing with a pattern stimulating only one polarity channel (Fig. 3B). The rVMP has been mostly observed when adaptation duration is less than 120 ms. The absence of rVMP effects in these conditions is probably due to the fact that the lowest adaptation duration (188 ms) used is too long for short-term facilitation, thus being overridden by a slower form of adaptation. On the other hand, when a test pattern probing the spatiotemporal correlation between two pathways was used, a significant rVMP was observed for both motion types. Such a difference in adaptation curves suggests that rVMP effects, for test patterns engaging across pathway correlation, are not restricted to short adaptation durations and may require relatively longer adaptation durations. Overall, these differences are in line with previous masking studies on contrast discrimination proposing that the processes requiring across pathway mechanisms are slower than the processes engaging only a single pathway^37^.

The perceptual sensitization (PS) was only observed when regular motion was used as adapting stimulus in Experiment 1. The perceived direction of regular motion is in the direction of physical displacement. Therefore, relative to reverse-phi motion, it has been generally considered that this type of motion may also engage higher-order attentive tracking in the direction of physical displacement^38^,^39^,^40^. The perceptual sensitization (PS) observed for regular motion in Experiment 1 might be due to the activation of attention-dependent motion mechanisms^21^,^24^,^27^,^28^. Future studies are required to understand how these relatively small contributions from other motion systems^41^,^42^ depend on adaptation duration and the test pattern used.

#### ON-OFF Pathway Signaling for Visual Motion Processing

Our results support the general view that the spatiotemporal correlation between ON and OFF pathways is essential to cover important aspects of low-level motion perception. In this respect, they are in agreement with models suggesting not only the presence of detection mechanisms within each pathway but also mechanisms combining information from both pathways at initial stages of motion processing. Two models in in this direction have been proposed by Mo *et al.*^17^ and Bours *et al.*^13^ Both of these models include low-level motion detectors sensitive to the same and opposite contrast polarity correlations. However, they differ in how the ON and OFF signals are combined. Accordingly, distinct characteristics of motion detectors in each model are identified. In the Mo *et al.*’s^17^ model, a motion detector sensitive to regular motion in one direction is equally activated by reverse-phi motion in the opposite direction. On the other hand, the model proposed by Bours *et al.*^13^ include detectors excited by regular motion in one direction, but inhibited by reverse-phi in the same direction. Based on Bours *et al.*^13^, at low-level motion detection, contrast reversals reverse the sign of the response rather than the direction. Recent evidence from human psychophysics and cell-recording from monkey V1 supports this excitation-inhibition scheme for the same and opposite contrast polarity correlations^13^,^16^. The behavioral results presented here suggest that these mechanisms driven by excitation and inhibition have distinct temporal dynamics.

#### Temporal Frequency Tuning

Recently, Pavan *et al.*^25^ reported that rMAE can only be generated by dynamic directionally ambiguous test patterns. By varying the temporal frequency of adapting and test patterns, they also investigated the temporal tuning characteristics of rMAE. Their findings highlight the involvement of both low- and high-pass visual channels in rMAE^43^,^44^,^45^,^46^. Furthermore, recent studies on the fly visual system report that reverse-phi motion is optimally perceived at lower temporal frequencies than those for regular motion^47^. In the present study, we only used a single temporal frequency yielding reliable regular and reverse-phi motion percepts. An interesting issue is whether there are differences on the involvement of temporal frequency channels for both motion types and whether they can be revealed using a rapid adaptation paradigm. Future studies systematically manipulating both spatial and temporal frequencies of adaptation and test patterns will be informative in this respect.

In conclusion, the present study demonstrates that spatiotemporal correlation within each pathway and across pathways can be selectively adapted by using regular and reverse-phi motion in a well-known rapid adaptation paradigm. We found that the signals from both pathways are effectively combined at initial stages of motion processing yielding short-term adaptation effects. As revealed by longer adaptation durations, the temporal characteristics of adaptation curves by stimuli mostly engaging spatiotemporal correlation between opposite polarities are different than those engaging correlation between the same polarities. Our findings support the presence of both within and across pathway mechanisms at early stages of visual motion processing.

## Additional Information

**How to cite this article**: Oluk, C. *et al.* Rapid Motion Adaptation Reveals the Temporal Dynamics of Spatiotemporal Correlation between ON and OFF Pathways. *Sci. Rep.* **6**, 34073; doi: 10.1038/srep34073 (2016).

## Supplementary Material

Supplementary Information

## Figures and Tables

**Table 1:** Corrected p-values for Experiment 1.

ISI	Regular Motion			Reverse-phi Motion		
	188 ms	376 ms	752 ms	188 ms	376 ms	752 ms
35 ms	0.4930	0.6350	***0***.0015*	0.2500	0.0912	***0***.0183*
118 ms	0.4012	0.5100	***0***.0015*	***0***.0200*	***0***.0175*	***0***.0045*
482 ms	0.4012	0.3050	***0***.0015*	***0***.0275*	***0***.0367*	***0***.0075*
1 s	0.4012	0.3050	0.4510	0.9420	***0***.0045*	0.2088
2 s	0.4012	0.3050	***0***.0037*	0.1950	0.4320	0.7240

One-sample t-tests were conducted to assess whether each condition was significantly different from the chance level. Multiple one-sample t-tests were corrected using a False Discovery Rate (FDR) at 0.05 for each adaptation condition^48^,^49^. Significant p-values (*p* < 0.05) are highlighted in bold and marked with an asterisk. Each row and column corresponds to ISI and adaptation duration conditions, respectively.

**Table 2:** Corrected p-values for Experiment 2.

ISI	Regular Motion			Reverse-phi Motion		
	188 ms	376 ms	752 ms	188 ms	376 ms	752 ms
35 ms	0.12000	0.0875	1.00000	***0***.0045*	0.6450	0.4000
118 ms	***0***.03500*	***0***.0100*	1.00000	***0***.0250*	0.7770	0.0975
482 ms	0.75000	0.8430	1.00000	0.8940	0.6450	***0***.0300*
1 s	1.00000	0.8430	1.00000	0.8940	0.3700	0.2550
2 s	0.75000	0.4717	1.00000	0.8940	0.7770	0.2067

We applied the same statistical approach as the one used for Experiment 1 (Table 1). Significant p-values (p < 0.05) are highlighted in bold and marked with an asterisk.
