# Supplementary material for: Rapid Motion Adaptation Reveals the Temporal Dynamics of Spatiotemporal Correlation between ON and OFF Pathways
Source: Sci Rep. 2016 Sep 26;6:34073. doi: 10.1038/srep34073 (PMC5036170; doi:10.1038/srep34073)
Supplement: Supplementary Information [file srep34073-s1.pdf]

Rapid Motion Adaptation Reveals the Temporal Dynamics of Spatiotemporal Correlation between ON and OFF Pathways

Can Oluk[1,3], Andrea Pavan[4], Hulusi Kafaligonul[1,2,*]

[1]National Magnetic Resonance Research Center (UMRAM), Bilkent University, Ankara, Turkey

[2]Interdisciplinary Neuroscience Program, Bilkent University, Ankara, Turkey

[3]Department of Psychology, Bilkent University, Ankara, Turkey

[4]University of Lincoln, School of Psychology, Brayford Pool, Lincoln, LN6 7TS, UK

SUPPLEMENTARY INFORMATION

Experiment 1

We analyzed the effect of contrast polarity by separating the bright and dark adapting conditions for *regular motion*. As shown in Table S1, the main effect of contrast polarity was not significant and the only significant interaction was with adaptation duration.

**Factor/Interaction**	**Degrees of freedom**	***F***	***p***	***partial-η²***
Contrast Polarity (bright/dark)	1,14	3.293	0.091	0.190
Adaptation Duration	2,28	19.469	0.000	0.582
ISI	4,56	8.512	0.000	0.378
Contrast Polarity x Adaptation Duration	2,28	4.310	*0.023*	*0.235*
Contrast Polarity x ISI	4,56	1.691	0.165	0.108
Adaptation Duration x ISI	8,112	12.293	0.000	0.468
Contrast Polarity x Adaptation Duration x ISI	8,112	0.621	0.758	0.043

Table S1. Three-way ANOVA output for regular motion adaptation conditions.

To disentangle the source of this two-way interaction, we conducted additional two-way ANOVA tests for each adaptation duration. The ANOVA reported only a significant effect of contrast polarity for the longest adaptation duration (**Table S2**: $F_{1,14} = 7.63$, $p = 0.015$, *partial-*$\eta^2 = 0.353$). Bonferroni corrected pairwise comparisons (*critical* $p = 0.024$) reported only a significant difference between bright and dark adapting stimulus when the ISI was 1 sec (**Figure S1**).

**Factor/Interaction**	**Degrees of freedom**	***F***	***p***	***partial-η²***
Contrast Polarity (bright/dark)	1,14	7.634	*0.015*	*0.353*
ISI	4,56	38.255	0.000	0.732
Contrast Polarity x ISI	4,56	0.830	0.512	0.056

Table S2. Two-way ANOVA results for the longest adaptation duration (752 ms) condition of regular motion.

The results for each contrast polarity of the longest adaptation condition (752 ms) is shown in Figure S1. Overall, the percentage of trials in which the test pattern was perceived to drift in the same direction to that of the bright adapting stimulus were higher than those obtained with the dark adapting stimulus. We also applied the same analysis and statistical approach on the motion direction reports from control sessions. The statistical tests did not reveal any significant interaction and main effect of contrast polarity.

Figure S1. The bright and dark adapting conditions of regular motion are separately shown for 752 ms adaptation duration. Error bars correspond to + SEM.

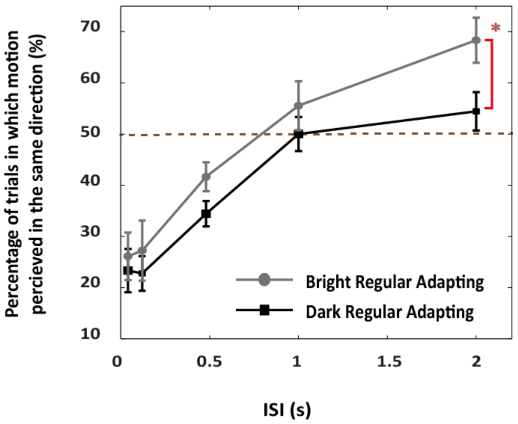

To test whether the effects of reverse-phi motion adaptation on the test pattern is dependent on the contrast polarity, we analyzed the bright and dark test pattern conditions of *reverse-phi* separately. There was only a significant interaction between contrast polarity and adaptation duration (**Table S3**).

Factor/Interaction	Degrees of freedom	*F*	*p*	*partial-η²*
Contrast Polarity (bright/dark)	1,14	0.215	0.650	0.015
Adaptation Duration	2,28	2.832	0.076	0.168
ISI	4,56	7.304	*0.000*	0.343
Contrast Polarity x Adaptation Duration	2,28	4.595	*0.019*	*0.247*
Contrast Polarity x ISI	4,56	1.625	0.181	0.104
Adaptation Duration x ISI	8,112	1.511	0.161	0.097
Contrast Polarity x Adaptation Duration x ISI	8,112	0.850	0.561	0.057

Table S3. Three-way ANOVA results for reverse-phi adaptation conditions. The contrast polarity condition was defined based on the polarity of test pattern.

We conducted two-way ANOVA tests for simple main effects to elucidate the nature of the significant interaction between contrast polarity and adaptation duration. We only found a significant interaction between contrast polarity and ISI for 376 ms adaptation duration (**Table S4**: $F_{4,56} = 2.74$, $p = 0.037$, $partial\text{-}\eta^2 = 0.164$).

Factor/Interaction	Degrees of freedom	*F*	*p*	*partial-η²*
Contrast Polarity (bright/dark)	1,14	2.633	0.125	0.160
ISI	4,56	2.451	0.056	0.149
Contrast Polarity x ISI	4,56	2.741	*0.037*	*0.164*

Table S4. Two-way ANOVA results for the 376 adaptation duration of reverse-phi motion. The contrast polarity condition was defined based on the polarity of test pattern.

However, further analyses on 376 ms adaptation duration condition did not report a systematic difference between bright and dark test patterns (**Figure S2**). Post-hoc Bonferroni corrected pairwise comparison (*critical* p = 0.024) indicated a significant difference between bright and dark test pattern only for 1 sec of ISI.

Figure S2. The bright and dark test pattern conditions of reverse-phi are separately shown for 376 ms adaptation duration. Error bars correspond to $\pm$ SEM.

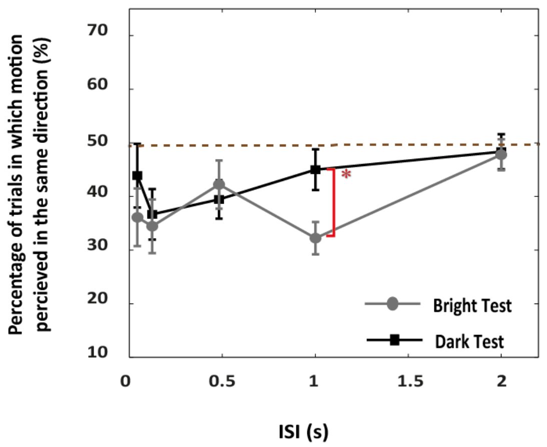

In our experiments, the starting phase of the test pattern (relative to the adapting stimulus) was not randomized. Therefore, this may lead to an apparent motion between the last frame of the adapting pattern and the first frame of test pattern for the shorter ISI values (e.g., 35 ms). If this is the case, the same and opposite polarity pairs of the last adaptation frame and the first test frame should result in regular and reverse-phi apparent motion, respectively. To assess this possibility, we analyzed the *reverse-phi motion* adaptation results by separating same and opposite polarity pairs. A three-way repeated measure ANOVA (the polarity pair, adaptation duration and ISI as factors) did not report a significant polarity pair effect ($F_{1,14}$ = 0.220, p = 0.660, *partial*-η^2 = 0.020) and only its interaction with adaptation duration was significant ($F_{2,28}$ = 4.590, p = 0.020, *partial*-η^2 = 0.250). Follow up two-way ANOVA tests on each adaptation duration revealed only a significant interaction between polarity pair and ISI ($F_{4,56}$ = 2.750, p = 0.040, *partial*-η^2 = 0.170) when adaptation duration was 376 ms. However, Bonferonni corrected pairwise comparisons did not report a significant difference at short ISI values.

Experiment 2

As in Experiment 1, we analyzed the effect of contrast polarity by separating the bright and dark adapting conditions of *regular motion*. The main effect of contrast polarity and its interactions with other factors were not significant (**Table S5**). Additionally, we analyzed the motion adaptation results by separating same and opposite contrast polarity pairs of the last adaptation frame and the first test frame. As in Experiment 1, we performed a three-way repeated measures ANOVA (the polarity pair, adaptation duration and ISI as factors) on each motion adaptation condition. For *regular motion* adaptation, the effect of polarity pair ($F_{1,11}$ = 1.501, p = 0.246, *partial*-η^2 = 0.120) and its interaction with adaptation duration ($F_{2,22}$ = 0.249, p = 0.782, *partial*-η^2 = 0.022) were not significant. However, the interaction between the polarity pair and ISI was significant ($F_{4,44}$ = 4.073, p = 0.007, *partial*-η^2 = 0.270). To understand the nature of this interaction, we performed two-way ANOVA tests for each ISI condition. We found significant effects of polarity pair for 35 ms ($F_{1,11}$ = 4.923, p = 0.048, *partial*-η^2 = 0.309) and 2 sec ($F_{1,11}$ = 11.000, p = 0.007, *partial*-η^2 = 0.500) ISI values. The small p value for 2 sec ISI condition suggests that this interaction was mostly due to the difference at this longest ISI.

For *reverse-phi* adaptation, three-way repeated measure ANOVA did not report a significant effect of polarity pair ($F_{1,11} = 0.560$, $p = 0.470$, *partial*-$\eta^2 = 0.048$). Moreover, its interaction with adaptation duration ($F_{2,22} = 0.768$, $p = 0.476$, *partial*-$\eta^2 = 0.065$), ISI ($F_{4,44} = 2.223$, $p = 0.082$, *partial*-$\eta^2 = 0.168$) and three-way interaction between all factors ($F_{8,88} = 1.410$, $p = 0.204$, *partial*-$\eta^2 = 0.114$) were not significant.

**Factor/Interaction**	**Degrees of freedom**	***F***	***p***	***partial*-η^2**
Contrast Polarity (bright/dark)	1,11	1.985	0.186	0.153
Adaptation Duration	2,22	4.220	0.028	0.277
ISI	4,44	6.322	0.000	0.365
Contrast Polarity x Adaptation Duration	2,22	0.490	0.619	0.043
Contrast Polarity x ISI	4,44	0.451	0.771	0.039
Adaptation Duration x ISI	8,88	2.174	0.037	0.165
Contrast Polarity x Adaptation Duration x ISI	8,88	0.824	0.584	0.070

Table S5. Three-way ANOVA results for regular motion adaptation conditions.
